# Rumen Microbiome Development in Lambs Following Maternal and Early-Life Prebiotic Mannan-Rich Fraction (MRF) Supplementation

**DOI:** 10.3390/ani16081137

**Published:** 2026-04-09

**Authors:** Aoife Corrigan, Stephen Stockdale, Alexander M. Mackenzie, Robert G. Wilkinson, Helen Warren, Jules Taylor-Pickard, Richard Murphy

**Affiliations:** 1 Alltech European Bioscience Centre, Dunboyne, A86 X006 Co. Meath, Ireland; rmurphy@alltech.com; 2 BioFigR, Ballyvoloon, Cobh, P24 N524 Cork, Ireland; stephen.stockdale@biofigr.com; 3 Department of Agriculture and Environment, Harper Adams University, Shropshire TF10 8NB, UK; amackenzie@harper-adams.ac.uk (A.M.M.); rgwilkinson@harper-adams.ac.uk (R.G.W.); 4 Alltech (UK) Ltd., Stamford PE9 1TZ, UK; hwarren@alltech.com (H.W.); jpickard@alltech.com (J.T.-P.)

**Keywords:** rumen microbiome, maternal supplementation, mannan-rich fraction, microbial resilience, metagenomics, volatile fatty acids, ewe, lamb, fetal programming, early-life programming

## Abstract

The early-life rumen microbiome is highly dynamic, shaped by dietary transitions and maternal influences. Several dietary additives have been studied during the pre- and post-weaning periods to improve animal welfare, growth performance, and farming efficiencies. This study investigated microbial community assembly and growth performance of lambs provided with a mannan-rich fraction (MRF) supplement, either through maternal supplementation, directly, or via a combination of both. Using metagenomic sequencing and gas chromatography, we found differences in rumen microbial alpha and beta diversity related to both sampling time point and MRF supplementation (*p* < 0.05). At week 8, lamb microbiomes showed greater variance in their Shannon alpha diversity, with direct MRF supplementation only to the lamb resulting in a significantly greater diversity (*p* < 0.05). At week 20, combined maternal and lamb supplementation resulted in the highest Shannon diversity and was different compared to all other groups (*p* < 0.05). Beta diversity analyses combined with differential abundance analyses revealed that microbial community structures are driven by both diet and time, with maternal MRF supplementation associated with enrichment of taxa involved in carbohydrate fermentation and succinate metabolism, including *Succiniclasticum ruminis*, *Succinovibrio dextrinosolvens*, and *Fibrobacter succinogenes*. Generalized linear modeling identified significant associations between microbial alpha diversity metrics and total volatile fatty acids in lambs, particularly butyrate and valerate. Furthermore, at week 8, there was a significant positive correlation between alpha diversity metrics and propionate and valerate. In this study, lambs receiving MRF through maternal and direct supplementation had the highest growth performance, measured as the median average daily gains (kg) and final weights (kg) of lambs. These findings suggest that MRF supplementation, especially when provided both maternally and directly, may influence the lamb rumen microbiome and alter its metabolic potential with potential implications for optimizing early-life nutrition strategies in ruminant production systems.

## Simple Summary

Early life is a critical period for lamb development, as rumen microorganisms help shape digestion, growth and overall health. This study looked at whether adding a natural yeast-based supplement to the diet of lambs, their mothers, or both could improve rumen microbial development and growth performance. The results showed that lambs receiving the supplement, especially when their mothers were also fed it, developed a more diverse and balanced microbial community in the rumen. The microbiome compositional shifts associated with supplementation also suggested a shift in fermentation pathways. While growth performance metrics did not differ significantly between dietary groups, lambs receiving both maternal and direct supplementation showed the highest median values for growth performance, suggesting potential biological relevance. Overall, these findings suggest that feeding this supplement during pregnancy and early life may influence rumen microbial development and fermentation patterns, with potential implications for digestive function and efficiency in sheep production systems.

## 1. Introduction

Rumen microbiota play a crucial role in ruminant health and productivity, influencing digestion, nutrient absorption, and overall performance [1,2]. The rumen microbiome, consisting of bacteria, protozoa, fungi, archaea, and viruses, forms a sophisticated network of symbiosis essential for the host maintenance, immune function, and overall production efficiency of the ruminant. This complex ecosystem enables ruminants to convert low-quality, fibrous plant material into usable energy and essential nutrients, a process that is not possible for non-ruminants [3].

A primary outcome of microbial fermentation is the production of volatile fatty acids (VFAs), primarily acetate, propionate, and butyrate. They serve as essential energy sources for the host animal, and can provide up to 70% of the total metabolizable energy for ruminants [4]. The interplay between the rumen microbiome and the host contributes to variation in many phenotypic traits expressed by the animal, including feed efficiency, milk composition, and growth performance [5]. Additionally, the rumen microbiome composition significantly impacts methane production, a potent greenhouse gas that contributes to the environmental footprint of livestock production [6].

The period from birth to weaning is critical for rumen microbial colonization and adaptation [7]. During this time, microbial communities are highly dynamic and influenced by factors such as diet composition, maternal nutrition, and environmental conditions, with effects that can persist into adulthood [8]. In this context, developmental programming provides a useful framework for understanding how early-life interventions shape long-term physiological and microbial outcomes [9].

Developmental programming in ruminants encompasses both fetal programming and early-life programming. Fetal programming refers to long-term effects on offspring physiology and metabolism that arise from environmental or nutritional conditions during gestation, before full development of the rumen and other organ systems [10]. Early-life programming encompasses nutritional and microbial exposures during the neonatal and pre-weaning period that shape the trajectory of rumen development, immune maturation, and microbial succession [11]. Recent reviews in ruminants highlight how maternal nutrition, placental function and perinatal environment program offspring growth, metabolism, and productivity through fetal programming mechanisms. Complementary work has emphasized that nutritional and microbial exposures during the neonatal and post-weaning period constitute early-life programming, with persistent effects on rumen function, methane emissions and efficiency [9,10,11,12,13,14]. In the present study, outcomes measured at 8 weeks of age primarily reflect fetal programming effects, as lambs are still in a pre-ruminant stage with limited rumen functionality and remain largely dependent on milk. In contrast, responses observed between 8 and 20 weeks represent a combination of fetal and early-life programming effects, coinciding with weaning, dietary transition, rumen maturation, and microbial stabilization. In lambs, the period from birth to weaning is also crucial from both an animal welfare and financial perspective, as early-life challenges can lead to increased mortality, reduced growth rates, and long-term productivity losses.

In recent years there has been a growing interest in the use of prebiotics to beneficially modulate the rumen ecosystem. Prebiotics, defined as a “substrate that is selectively utilized by host microorganisms conferring a health benefit”, have shown promise in modulating rumen ecosystems [15,16]. Among these, mannan-rich fraction (MRF), a yeast-derived prebiotic, has gained attention for its potential to beneficially influence rumen microbial communities during early development. These compounds are not digested by the host but are selectively utilized by beneficial rumen microbes, promoting their growth and metabolic activity.

Prebiotics may modulate the rumen ecosystem by selectively stimulating microbial taxa involved in carbohydrate degradation, cross-feeding, and short-chain fatty acid production, thereby altering both community structure and fermentation function [17,18,19]. Metagenomic studies in ruminants suggest that such interventions can enrich taxa linked to fiber and starch utilization and shift functional pathways related to fermentation end-products, including propionate-associated metabolism and methane-linked microbial processes [20]. These compositional changes are often accompanied by altered volatile fatty acid production, especially increased total VFA concentration and changes in acetate, propionate, and butyrate proportions, indicating a shift in rumen energy partitioning [17,21]. In early-life ruminants, these effects may be particularly important because the rumen microbiome is still developing and is highly responsive to nutritional programming.

While prebiotics have been extensively studied in monogastric animals, the application of these prebiotics in ruminants, such as in ewe–lamb systems, remains relatively underexplored [22]. Recent work in dairy goat kids showed that early-life MRF supplementation elevated ruminal acetate and total volatile fatty acids, while also increasing the abundance of *Bacteroidetes* and *Succinivibrio* and decreasing *Firmicutes* and *Succiniclasticum*, demonstrating coordinated shifts in microbial composition and fermentation output [17,23,24]. Together, these findings suggest that prebiotic supplementation may shape both the taxonomic and functional maturation of the rumen microbiome, with potential implications for nutrient utilization, growth performance, and methane-related fermentation pathways.

Despite increasing interest in developmental programming in ruminants, several key knowledge gaps persist. These include limited integration of fetal and early-life programming, poor understanding of maternal effects on postnatal rumen development, a lack of longitudinal links between early colonization and functional outcomes, and limited insight into how maternal and direct interventions interact to shape microbial succession and metabolism.

Therefore, this study aimed to investigate the effects of maternal and early-life supplementation with a mannan-rich fraction (MRF) within a developmental programming framework. Specifically, maternal supplementation during gestation and lactation represents a fetal programming intervention, while direct supplementation during the pre- and post-weaning period represents an early-life programming intervention. Using metagenomic sequencing and gas chromatography, we assessed (1) microbial community structure and diversity, (2) taxonomic shifts linked to prebiotic exposure, and (3) VFA production profiles in lambs at critical developmental stages. We hypothesized that maternal, direct and combined prebiotic supplementation would differentially influence lamb rumen microbial development, fermentation profiles, and growth performance.

## 2. Materials and Methods

### 2.1. Animals, Diets and Experimental Design

The experiment was conducted at Harper Adams University between January and April 2021 in the Agriculture and Environment Department animal project buildings and was undertaken in accordance with the United Kingdom Animals (Scientific Procedures) Act, 1986 (amended 2012), and received local ethical approval. Forty-eight twin-bearing (confirmed by ultrasound scanning) Suffolk × Mule ewes from the Harper Adams University flock were housed individually from day 105 (week −6) of gestation and allocated to one of two groups in a randomized block design, blocked by parity, live weight and body condition score. Each dietary group comprised twenty-four replicates. The dietary groups consisted of either a control diet comprising straw and concentrates or an MRF-supplemented group, which received the same control diet with MRF added to the concentrate at 1 kg/t (manufacturer’s recommended inclusion rate). The mannan-rich fraction was supplied by Alltech Inc. (Nicholasville, KY, USA), and the ewe and lamb concentrates were manufactured by HJ Lea Oakes Ltd., Nantwich, Cheshire, UK (Table 1).

**Figure 1:**
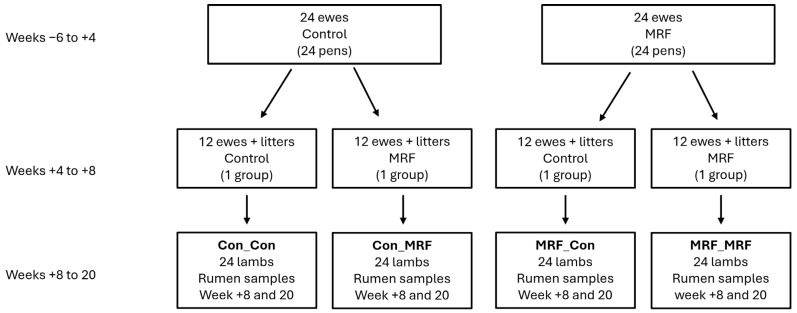
Trial design for study investigating the effect of MRF supplementation in ewes and/or lambs on the lamb rumen microbiome.

All ewes were housed individually on sawdust from week −6 of gestation to week +4 of lactation, with a mean lambing date of mid-February. Straw was coarsely chopped and offered *ad libitum*, with concentrates fed to provide a rising plane of nutrition to meet the metabolizable energy and metabolizable protein requirements of twin-bearing ewes during late pregnancy and producing 3.0 liters of milk during early lactation [25]. From week +4 of lactation, ewes and lambs on each treatment were allocated by block into two groups (12 ewes per group), group-housed and maintained on their experimental diets until week +8. This produced four treatment groups based on maternal diet (Control or MRF) and direct lamb supplementation (Control or MRF): Con_Con (control ewe, control lamb), Con_MRF (control ewe, MRF lamb), MRF_Con (MRF ewe, control lamb), and MRF_MRF (MRF ewe, MRF lamb). From week +4, suckling lambs were offered creep feed containing either no MRF (Control) or 1.0 g/kg MRF (MRF) (Figure 1). Lambs were weaned at week +8 and were individually penned on sawdust and offered their respective creep feeds *ad libitum* with a small amount of straw in racks. Due to a variety of factors unrelated to the experimental treatments, 11 ewes (6 control and 5 MRF-supplemented) and their lambs were removed from the study throughout the experiment.

### 2.2. Lamb Performance and Rumen Sampling

Following group housing at week +4, lamb creep intake was monitored on a group basis up until weaning at week +8. Individual intake could not be measured under these conditions; however, all lambs had equal access to creep feed within treatment groups. Following weaning, lambs were individually housed and offered their respective creep diets. Individual lamb feed intake was monitored by weighing the amount offered daily and weighing back refusals twice weekly. All intake values were adjusted for dry matter content. Lamb weight was recorded at birth and weekly at a fixed time relative to morning feeding to minimize variation due to gut fill. Following weaning at week +8 and at 42 kg liveweight (approximately week +20), one lamb from each set of twins was randomly selected for slaughter at a commercial slaughterhouse using a random number generator, ensuring equal probability of selection. At week +8 lambs (*n* = 37) were slaughtered across the four treatment groups: Control + Control (*n* = 8), Control + MRF (*n* = 9), MRF + Control (*n* = 10), and MRF + MRF (*n* = 10). At week +20, lambs (*n* = 36) were slaughtered, with group sizes as follows: Control + Control (*n* = 7), Control + MRF (*n* = 9), MRF + Control (*n* = 10), and MRF + MRF (*n* = 10). During slaughter, samples of rumen fluid were collected for VFA analysis, and samples of rumen fluid and digesta were collected for microbiome analysis as follows: subsamples of rumen digesta were filtered through two layers of muslin into a 100 mL sample pot. For VFA analysis 8 mL of rumen fluid was then pipetted into 15 mL tubes containing 2 mL of metaphosphoric acid and stored at −20 °C prior to further analysis. For microbiome analysis, 3 mL rumen fluid and 0.5 g of rumen digesta were transferred into tubes containing 5 mL DNA Shield (Zymo Research, Irvine, CA, USA).

### 2.3. VFA Analysis

Rumen fluid VFA concentrations were determined by gas chromatography (GC) using procedures described by Johnson et al. (2023) [26]. The GC was fitted with a DB-FFAP column (30 m × 0.250 mm × 0.2 µm; Agilent J and W, GC columns, Cheadle, UK) and a flame ionization detector (Agilent Inc., Wilmington, DE, USA). Conditions were: carrier gas, nitrogen; flow rate, 2.7 mL/min; column pressure, 0.8 bar; split ratio, 30:1; oven temperature, 235 °C; injector temperature, 250 °C; and detector temperature, 300 °C.

### 2.4. DNA Extraction, Library Construction, Quality Control, Sequencing and Bioinformatic Processing

DNA was extracted from rumen contents using a ZymoBIOMICS DNA Miniprep Kit (Zymo Research, Irvine, CA, USA) according to the manufacturer’s instructions. The genomic DNA concentration, purity, and integrity were determined using an Agilent 5400 Fragment Analyzer System (Agilent Technologies, Santa Clara, CA, USA). Sequencing libraries were generated using Genomic Nextera XT library preparation for Illumina (NEB, Ipswich, MA, USA) (Baseclear, Leiden, The Netherlands). The library preparations were sequenced on an Illumina NovaSeq 6000 platform, using an S4 flow cell generating 5 GB paired-end 150 bp reads per sample (Illumina, San Diego, CA, USA). Raw FASTQ files were produced using bcl2fastq (v2.20, Illumina). Initial quality filtering was performed using Illumina Chastity filtering to remove low-quality reads. Subsequently, reads containing PhiX control sequences were removed, and adapter sequences were trimmed, retaining reads with a minimum length of 50 bp. Quality of the filtered reads was assessed using FastQC (v0.11.8). Only high-quality reads passing these filtering steps were retained for downstream analysis. As the objective of this study was taxonomic profiling rather than genome reconstruction, de novo assembly was not performed. High-quality reads were directly subjected to taxonomic classification using Kraken2 (v2.1.1) against a curated reference database [27].

### 2.5. Raw Data Processing

Taxonomic classification outputs generated using Kraken2 were provided as raw count tables containing read assignments at multiple taxonomic levels for each sample. These data and associated metadata files were converted to CSV format and imported into R Studio (v.2024.04.02) for downstream processing [28]. Sample names and metadata variables were standardized, and the content of data frames was reviewed with the aid of the summarytools package (v1.0.1) [29]. A duplicate animal code was identified as associated with two VFA profiles; therefore, the relevant VFA data for those two time points were excluded from analyses. Missing values for initial birth weight, final weight, average daily gain, or total gain were identified where values could not be calculated from recorded data. To retain balanced statistical comparisons, missing values were replaced with an R-generated random number, using the mean and standard deviation for the respective group at the relevant time point. This approach was applied sparingly to avoid inconsistent sample inclusion across analyses. Prior to statistical testing, data were assessed for normality using the Shapiro–Wilk test.

### 2.6. Count Data Processing

Taxonomic names in the microbiome count tables were cleaned of non-alphanumeric symbols. Where species information was known but higher taxonomic ranks were recorded as NA, missing values were replaced with taxonomic information retrieved from publicly available databases. When working with taxonomic ranks above species, and species converged on a higher taxon, all count data for duplicated row names were aggregated. A Caudoviricetes virus, an arbuscular mycorrhizal fungus, and a non-ovine vertebrate taxonomic entry were removed from the count matrices. After confirming the percentage of reads assigned as Unclassified Bracken and Unclassified Kraken2 were not statistically different (Wilcoxon test, *p* > 0.05) among dietary groups or time points, unclassified reads that would be biologically uninformative were removed from the final, cleaned microbiome count data.

### 2.7. Microbiome Diversity Analysis

Microbiome count matrices were manipulated as required using dplyr (v1.1.4) [30] and tidyr (v1.3.1) [31] syntax, but with long-to-wide data frame format changes performed using the reshape2 package (v1.4.4) [32]. Microbiome intra-sample alpha diversity and inter-sample beta diversity calculations were generated using the vegan (v2.6.8) [33] and phyloseq (v1.48.0) [34] packages. Beta diversity differences were calculated using the Bray–Curtis ecological distance metric, with principal coordinate analysis (PCoA) ordination. The plot_scree function of phyloseq was used to calculate Eigenvalues. The Permutational Multivariate Analysis of Variance test (PERMANOVA) was used to assess whether individual and interactive effects of metadata variables explained the observed variation in the beta diversity data. Furthermore, Analysis of Similarities tests (ANOSIM) were used on subsets to evaluate ranked Bray–Curtis dissimilarities.

### 2.8. Additional Microbiome Analyses

Compositional differences in microbial taxa were determined using Analysis of Compositions of Microbiomes with Bias Correction 2 (ANCOM-BC2) (v2.6.0) [35]. Simplified putative roles were assigned to ruminant taxa for illustrative purposes, using OpenAI’s 4o model [36] with manual review. The microbial interaction network was generated using SpiecEasi with the Meinshausen–Bühlmann (mb) graphical lasso method (settings: lambda.min.ratio = 1 × 10^−3^, nlambda = 20) (v1.1.3) [37]. Relative abundances were calculated using the funrar package (v1.5.0) [38], whereas centered log-ratio (CLR) transformations were performed by adding +1 to count values, dividing these non-zero count values by the sum of reads across each relevant sample, and finally applying the SpiecEasi CLR function.

### 2.9. Statistical Testing Approaches

The Shapiro–Wilks test with histogram visualization using base R was used to assess distribution and normality of data. Levene’s test from the car package (v3.1.3) [39] was used to assess variance. T-tests were applied to determine statistical significance between two normally distributed groups. Wilcoxon and Kruskal–Wallis tests were used for two-group and three-or-more-group statistical tests, respectively, when data distribution was found to be non-parametric. Where necessary, Benjamini–Hochberg false discovery rate corrections for multiple comparisons were performed. The log fold-change and *p*-values associated with microbial compositional differences were calculated using ANCOM-BC2, with default settings. Pearson and Spearman correlations from the Psych R package (v2.4.6.26) [40] were used for normally distributed and non-parametric tests, respectively. Gaussian-distributed Generalized Linear Models (GLMs) were generated using the stats base package of R. A Gaussian distribution model was selected based on comparison of Akaike Information Criterion values across candidate distributions, including Gamma and inverse Gaussian. Only one liquid-phase sample per animal (paired with VFA data) was used to avoid repeated measures. Animal lineage and pen assignment data were not available during modeling and were therefore not included as covariates.

### 2.10. Figure Generation Procedures

Plots were generated using ggplot2 (v3.5.1) [41] with additional statistical and plotting functions supported through the ggpubr (v0.6.0) [42] and cowplot (v1.1.3) [43] packages. Data point labels on the network plot and volcano plots were supported using the ggrepel package (v0.9.6) [44]. The igraph package with the Fruchterman–Reingold (fr) layout [45] was implemented to finalize the microbiome network interaction plot. Additional colors were supported using the RColorBrewer package (v1.1-3) [46]. Manual edits to create the final quality images were made using Inkscape (version 1.3).

## 3. Results

### 3.1. Overview of the Lamb Rumen Microbiome

**Figure 2:**
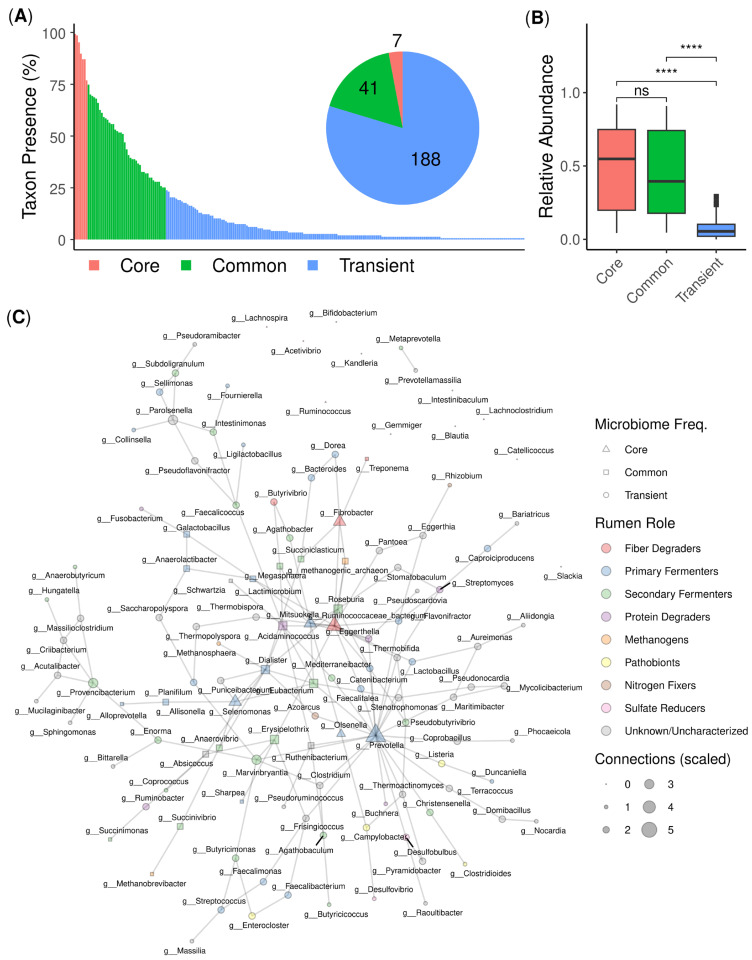
Lamb rumen microbiome. (**A**) Presence of all genera detected across all shotgun metagenome samples (*N* = 146), illustrated as core (>75%; *n* = 7), common (≤75% and ≥25%; *n* = 41), and transient (<25%; *n* = 188) taxa. (**B**) The relative abundance of core, common, and transient genera per sample. Wilcoxon test legend: **** < 0.0001, ns = not significant. (**C**) SpiecEasi microbiome network interaction plot, with a putative role within rumens assigned for graphical representation. The size of each node is scaled using the formula: log2(edges + 1).

In this study, we analyzed the lamb rumen microbiome at two time points, week 8 and week 20 after birth. Week 8 represents an important period in lamb development due to weaning and a transition to foraging diets. Across 146 shotgun metagenome samples from both time points, only seven core microbial genera were detected (i.e., genera present in more than 75% of samples) (Figure 2A). A conservative prevalence threshold (>75%) was selected to identify the most consistently detected taxa during a highly dynamic developmental period. In contrast, most of the genera (79.7%, or 188 out of 236) were classified as transient, being present in fewer than 25% of samples. Genera present in 25–75% of samples were defined as common.

Core and common genera detected exhibited significantly greater relative abundance than transient taxa (Figure 2B). Despite their lower prevalence, the importance of transient or functionally redundant taxa is highlighted in a microbial network (Figure 2C), where many of these taxa were frequently annotated as primary and secondary fermenters. These taxa showed frequent connections within the co-occurrence and putative interaction network, suggesting potential functional relevance despite their low prevalence. As expected, functionally diverse taxa involved in early fiber degradation and fermentation processes, such as *Prevotella* and *Ruminococcaceae*, had the most edges in the network plot, with 23 and 16 connections, respectively.

### 3.2. Alpha Diversity Differences by Diet and Time

**Figure 3:**
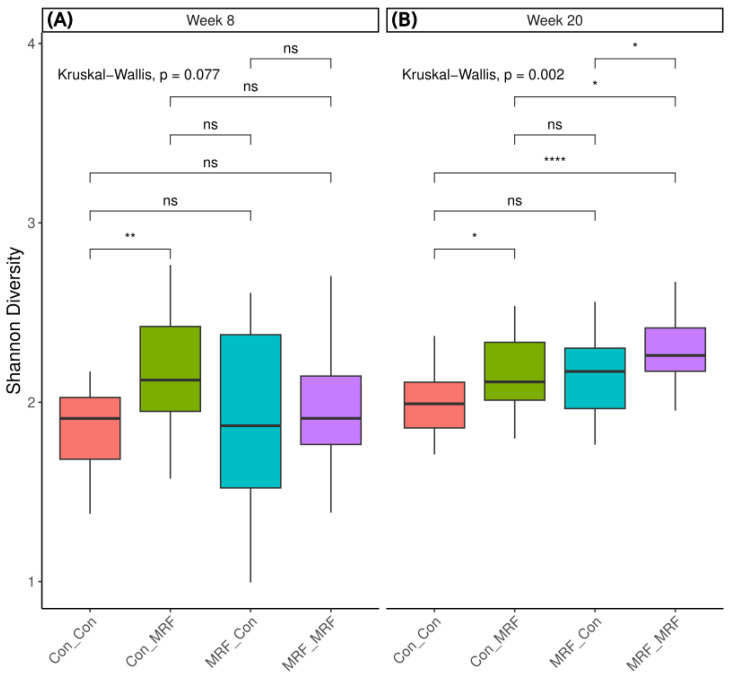
Alpha diversity analysis. Microbiome intra-sample diversity analyzed using the Shannon index diversity metric, separated by (**A**) Week 8 and (**B**) Week 20. Wilcoxon tests were performed among groups, while Kruskal–Wallis tests were performed across groups. Wilcoxon test legend: * < 0.05, ** < 0.01, **** < 0.0001, ns = not significant.

**Figure 4:**
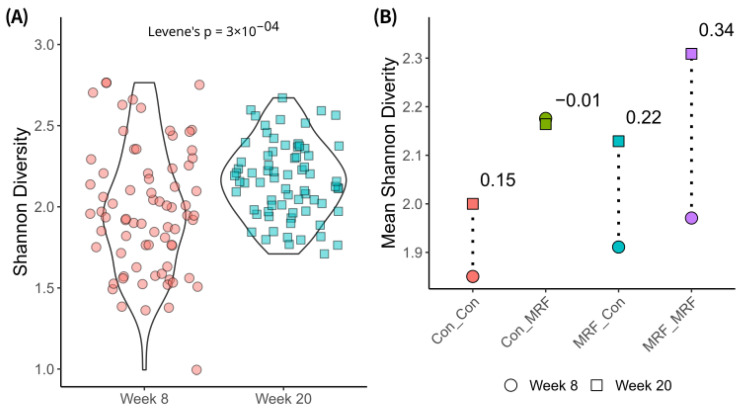
Loss of alpha diversity variance over time. (**A**) Distribution of Shannon diversity values across all samples at week 8 and week 20. Levene’s test was used to assess whether variability in alpha diversity differs between time points, with the *p*-value shown. (**B**) Grouped mean Shannon diversity values for each diet cohort at weeks 8 and 20. Inset numbers indicate the numeric change in group mean from week 8 to 20. This panel allows comparison of central tendencies across groups over time.

To assess differences in the lamb microbiome, including those associated with direct lamb-fed MRF supplementation, and with or without maternal dietary MRF supplementation, we analyzed alpha diversity metrics using Shannon, Simpson and Chao1 indices. For Shannon index alpha diversity, at week 8, only lambs fed MRF (Con_MRF) differed significantly from the control diet (Con_Con; Figure 3A). There was significantly more variance in Shannon diversity values among the groups at week 8 than at week 20 (Figure 4A).

At week 20, there was less variance among alpha diversities (Figure 4A), and there were statistically significant differences in lamb rumen microbiome alpha diversities (Figure 3B), indicating that both direct lamb and maternal MRF supplementation influenced alpha diversity. Specifically, compared to the control group (Con_Con), lambs that received MRF either directly only (Con_MRF) or in both the lamb and mother’s diet (MRF_MRF) had significantly higher alpha diversity. When both lambs and their mothers received MRF (MRF_MRF), alpha diversity was significantly greater than all other groups (Con_Con, Con_MRF, and MRF_Con). Looking at intra-diet comparisons, between week 8 and week 20 time points, the greatest difference in mean alpha diversities was observed when lambs and mothers were both supplemented with MRF (MRF_MRF; Figure 4B). Differences in lamb rumen microbiomes with dietary and maternal MRF followed similar trends to the Shannon index when analyzed using either the Simpson or Chao1 indices.

### 3.3. Beta Diversity Differences by Diet and Time

**Figure 5:**
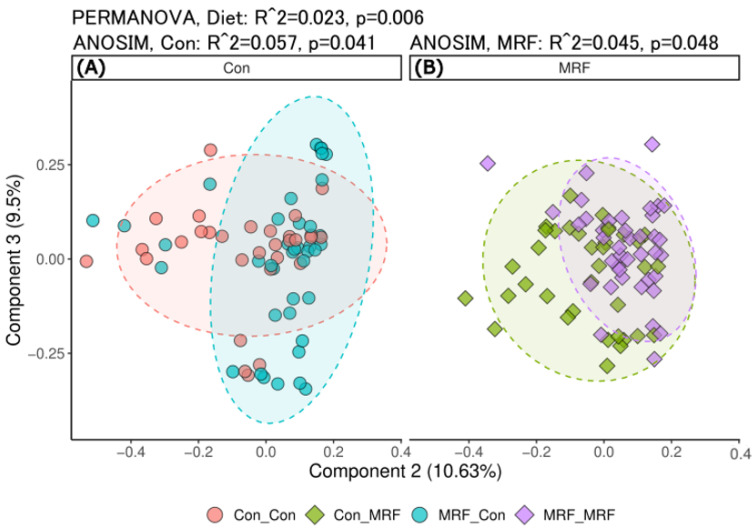
Beta diversity comparison of lamb microbiota by maternal and postnatal diet. PCoA ordination of Bray–Curtis distances showing microbiome dissimilarity in lambs, separated by postnatal diet group (Con or MRF). (**A**) Lambs on the control diet, comparing those born to control-fed vs. MRF-fed ewes. (**B**) Lambs on the MRF diet, comparing maternal diet groups. PERMANOVA was used to test for global effects of diet (R^2^ = 0.04561, *p* = 0.002), while pairwise ANOSIM tests assessed beta diversity differences within each postnatal diet group. Principal coordinate axes two and three are shown.

To investigate differences in microbiome composition among dietary groups, we compared beta diversity using Bray–Curtis dissimilarities and principal coordinate analysis (PCoA). Across all samples, time was the primary driver of community separation. By Eigenvalue, the separation of samples by principal component one (PC1) was 45.6%, and a Spearman correlation of the PCoA ordination coordinates of PC1 versus time showed a significant association (rho −0.46, *p* = 7.7 × 10^−9^). In contrast, the ordination of samples on PC2 and PC3 was less influenced by time (rho 0.109 and −0.002, respectively). Dietary effects were primarily observed along PC2 (rho 0.36, *p* = 7.6 × 10^−6^). PERMANOVA analysis confirmed that both time and diet significantly explained variation in microbiome composition (R^2^ = 0.046 and 0.023; *p* = 0.002 and 0.006, respectively). Focusing on the influence of maternal MRF supplementation, we examined Bray–Curtis PCoA plots for lambs either receiving (Con_MRF v MRF_MRF) or not receiving (Con_Con v MRF_Con) MRF directly (Figure 5). A focused ANOSIM comparison of the lambs without MRF but differing in maternal MRF supplementation (Con_Con and MRF_Con) had a greater variance explained, as shown by a higher ANOSIM R^2^ (Figure 5A, R^2^ = 0.057) than lambs on MRF and differing in maternal MRF supplementation (Con_MRF and MRF_MRF; Figure 5B, R^2^ = 0.045). The greater variance associated with the lambs without MRF indicates that maternal MRF exposure can influence microbiome development, particularly in the absence of direct postnatal MRF supplementation.

### 3.4. Lamb Rumen Microbiome Diversity and Total Volatile Fatty Acids (TVFAs)

**Figure 6:**
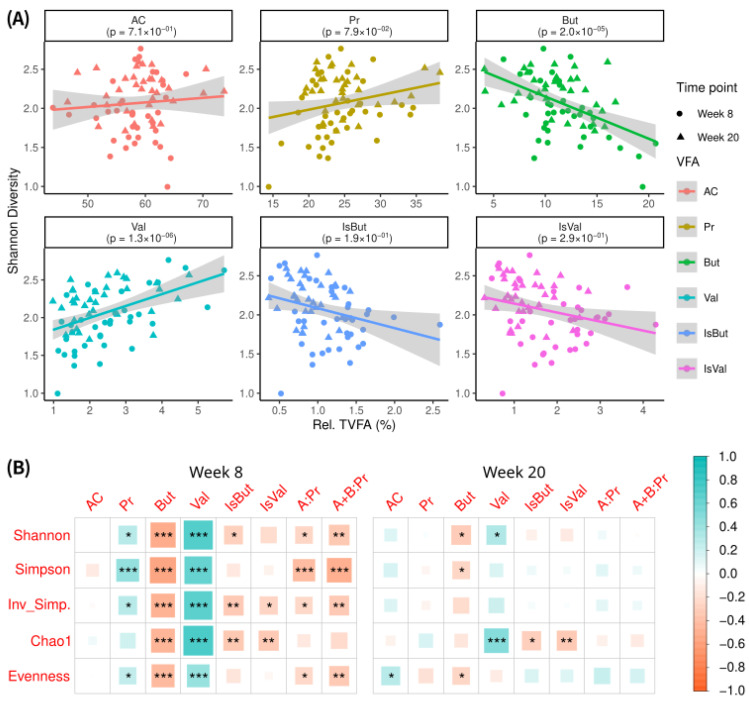
Alpha diversity and TVFAs. (**A**) Gaussian-distributed generalized linear model (GLM) investigating whether Shannon alpha diversity explains the variation observed in the relative proportions of short-chain fatty acids. (**B**) Pearson correlations of alpha diversities versus the relative total volatile fatty acid (TVFA) percentage of samples at week 8 and week 20. Correlation legend: * < 0.05, ** < 0.01, *** < 0.001, A: Pr = acetate to propionate ratio, A + B: Pr = acetate and butyrate to propionate ratio.

To assess the functional implications of MRF-induced microbiome shifts, we examined the relationship between alpha diversity (Shannon index) and total volatile fatty acids (TVFAs). TVFA can be broken into short-chain fatty acids (SCFAs), acetate, propionate, butyrate, and valerate, and the branched-chain fatty acids (BCFAs) of isobutyrate and isovalerate. SCFA and BCFA values were normalized as proportions of total TVFAs to account for inter-lamb variation. Across all liquid metagenome samples matched to a VFA sample, where time was included as a fixed effect in a generalized linear model (GLM), alpha diversity showed a significant positive association with valerate and a negative association with butyrate (Figure 6A). The results and trends were consistent when the GLM was run per individual time point (Table 2). When Pearson correlations were performed between each liquid sample’s alpha diversity and its TVFA, separated by time point, week 8 showed significant positive correlations with propionate and valerate, and significant negative correlations with butyrate, isobutyrate, acetate:propionate, and acetate + butyrate:propionate (Figure 6B). There were fewer week 20 correlations between alpha diversity and TVFAs, and no association was observed between alpha diversity and acetate at either time point. However, the trends and statistically significant observations at week 20 were consistent with those observed at week 8.

### 3.5. Microbiome Compositional Differences Associated with MRF

**Figure 7:**
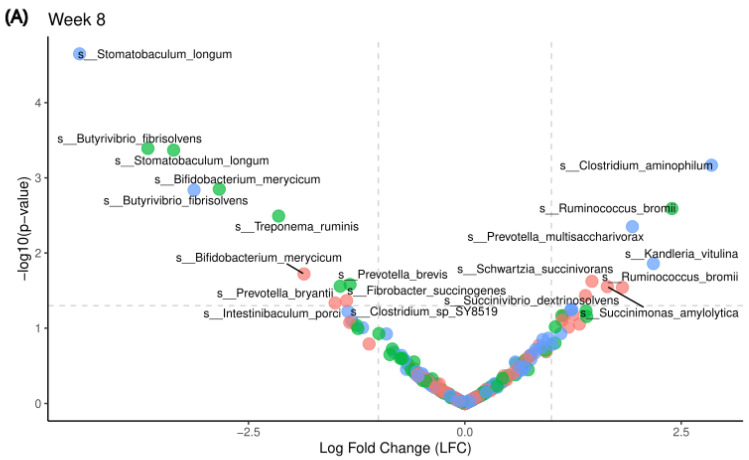

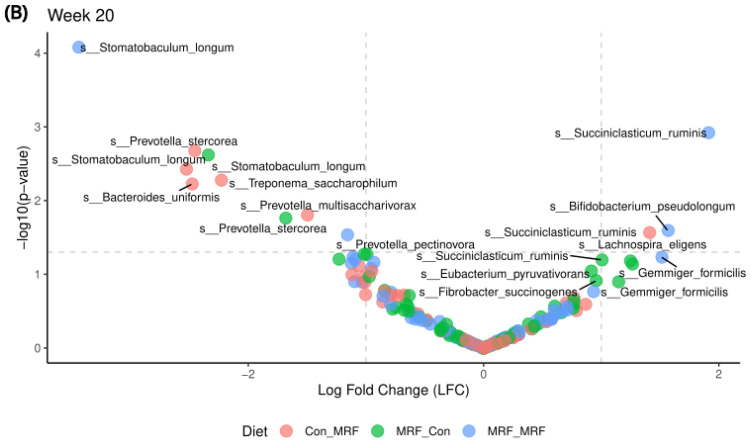
Microbiome compositional differences. ANCOM-BC2 analyses the compositional differences in diets at (**A**) week 8 and (**B**) week 20 relative to the control diet, Con_Con. The vertical grey dashed lines represent a log fold-change of 1.0, whereas the horizontal dashed line is set at 1.3, which represents a *p*-value = 0.05.

To determine differences in microbiome composition among MRF supplementation groups compared with the Control (Con_Con), ANCOM-BC2 was used to calculate diet- induced differences at week 8 and week 20 (Figure 7). Across dietary conditions, we observed that the addition of the MRF supplement altered taxa involved in succinate metabolism. While there are redundant taxa capable of producing and consuming succinate, we observed that MRF-supplemented diets had greater abundances of taxa such as *Succinovibrio dextrinosolvens* (week 8), *Succinomonas amylolytica* (week 8), *Schwartzia succinivorans* (week 8), and *Fibrobacter succinogenes* (week 20). *Succiniclasticum ruminis* (week 20) was more abundant across all MRF-supplemented diets relative to the Control diet (Con_Con).

**Figure 8:**
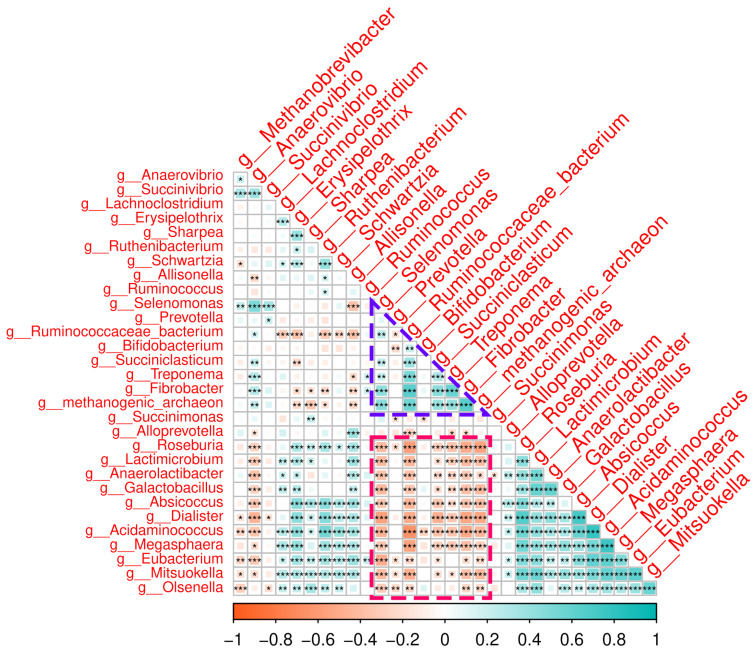
Taxonomic correlation analysis. Spearman correlation analysis of all core and common genera detected across all samples, using the centered log ratio (CLR) abundances of taxa. Two groups are highlighted for clarity. (1) Purple triangle: a co-occurring (positively correlating) group of succinate-fermenting genera. (2) Pink rectangle: a group of lactate acid producers that negatively correlate with succinate fermenters. Correlation legend: * < 0.05, ** < 0.01, *** < 0.001.

To further explore potential functional interactions, we examined co-occurrence patterns among taxa via correlation analysis. *Prevotella*, a core microbiome taxon, infrequently correlated with other taxa (Figure 8); this result supports their predicted role within the rumen as a genus involved in diverse metabolic processes. In contrast, two distinct groups of co-occurring taxa emerged. *Ruminococcacaeae_bacterium* and *Fibrobacter* (succinate producers) positively correlated with *Selenomonas* (a succinate consumer). This small group of succinate-metabolizing genera (Figure 8, purple highlight) negatively correlated with a much larger group of taxa putatively involved in lactate and amino acid metabolism (Figure 8, pink highlight). This second group of taxa includes the core taxon *Mitsuokella* and *Lactomicrobium* (lactate producers), *Megasphaera* and *Dialister* (lactate consumers), and *Acidaminococcus* and *Eubacterium* (amino acid consumers). These contrasting clusters suggest potential metabolic partitioning within the rumen, with MRF supplementation favoring succinate-associated pathways.

### 3.6. Lamb Weight Gains with MRF Supplementation

**Figure 9:**
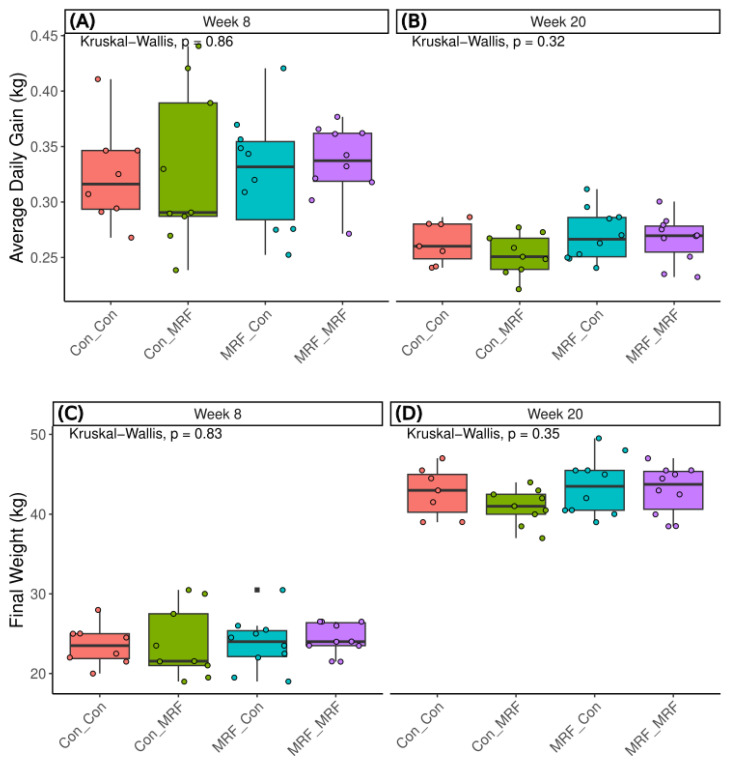
Lamb weight gains. Contrasting the average daily weight gain (kg) of lambs at (**A**) week 8 versus (**B**) week 20 across dietary groups. Highlighting the final weights (kg) of lambs in different dietary groups at (**C**) week 8 and (**D**) week 20. Average daily gains were calculated as the respective weights at week 8 and week 20, relative to 56 or 140 days, respectively. Kruskal–Wallis test results are inset within the corresponding panel.

Dietary group comparisons showed no statistical difference in week 8 or week 20 average daily gain (ADG) or final weight (Figure 9). As shown in the boxplots, lambs receiving MRF in both the maternal and early-life diets (MRF_MRF) exhibited the highest median average daily gain (ADG) and final body weight across treatment groups. Specifically, the MRF_MRF group showed the highest median ADG at week 8 (0.34 kg d^−1^) compared with Con_Con (0.32 kg d^−1^), Con_MRF (0.29 kg d^−1^), and MRF_Con (0.33 kg d^−1^), and maintained a higher median ADG at week 20 (0.27 kg d^−1^) relative to Con_Con (0.26 kg d^−1^), Con_MRF (0.25 kg d^−1^), and MRF_Con (0.27 kg d^−1^). Similarly, the MRF_MRF group achieved the highest median final body weight at week 8 (24.00 kg) compared with Con_Con (23.50 kg), Con_MRF (21.55 kg), and MRF_Con (24.00 kg), and at week 20 (43.75 kg) compared with Con_Con (43.0 kg), Con_MRF (41.0 kg), and MRF_Con (43.5 kg).

## 4. Discussion

During early life, microbial colonization of the mammalian digestive tract is a dynamic process and plays a key role in host health and development [7,47]. In young ruminants, during the early stages of development before the rumen is fully functional, the digestive system operates similarly to that of non-ruminant animals [47,48]. The mature rumen becomes colonized by a dense and diverse microbiota, composed of bacteria, archaea, protozoa and fungi, which degrade and ferment ingested feed [49]. The lamb rumen microbiome undergoes significant compositional shifts during early developmental stages [50]. During the most critical transition period, coinciding with dietary change to forage-based nutrition, microbial communities exhibited low stability or high interpersonal variability, with only seven core taxa persisting in >75% of samples (Figure 2A). A conservative prevalence threshold (>75%) was selected to identify the most consistently detected taxa during this highly dynamic developmental period, consistent with previous rumen microbiome studies that have applied stringent prevalence cutoffs to define core microbial taxa [51,52,53]. Although these core and common genera represented the majority of microbial abundance (Figure 2B), the limited taxonomic conservation (seven core taxa) suggests a small essential community of microbes. These dominant core taxa, including members of the *Ruminococcaceae* family and the genus *Prevotella*, are well recognized for their roles in ruminal carbohydrate fermentation. In particular, *Ruminococcaceae* contribute to the degradation of structural plant polysaccharides, while *Prevotella* are involved in the utilization of a broad range of carbohydrates and fermentation intermediates. These taxa likely support key rumen functions, including pH regulation and metabolite exchange, by breaking down complex carbohydrates and producing volatile fatty acids (VFAs) [54,55,56].

In contrast, transient or functionally redundant taxa that were not conservatively maintained between time points emerged as critical ecological players, with 43% annotated as primary/secondary fermenters (Figure 2C). This community structure, where numerically dominant core taxa coexist with a functional reservoir, may represent an adaptive strategy for developing ruminants. The interaction network further supports this structure, indicating that *Ruminococcaceae* and *Prevotella* act as central nodes linking core and transient taxa [54]. During perturbations to the rumen, such as diet changes, the rumen likely becomes more open to temporary microbes that have specialized enzymes for breaking down new types of plant material [56]. As these microbes join the community, they may help the rumen adjust to the new diet. At the same time, core bacteria like the *Ruminococcaceae* and *Prevotella* likely continue to support energy production by consistently breaking down carbohydrates.

Our study indicates that both time points and dietary supplementation with mannan-rich fraction (MRF) significantly shaped the alpha and beta diversity of the lamb rumen microbiome. During the critical window of rumen development at week 8, only lambs receiving direct MRF supplementation without maternal supplementation (Con_MRF) exhibited a significantly greater Shannon diversity, and variance in alpha diversity was also higher at this early stage. This higher variance may reflect the dynamic restructuring of the rumen microbiome during the dietary transition from milk to solid feed, when high microbial plasticity and stochastic colonization events can set the foundation for future resilience or vulnerability in later life [57,58].

At week 20, the synergistic effect of both maternal and lamb MRF supplementation became evident, with the MRF_MRF group also showing the greatest change in alpha diversity between the two time points. This underscores the potential for persistent dietary interventions to impact microbiome diversity and function and suggests a synergistic effect of combined maternal and lamb supplementation. This aligns with previous reports that early-life dietary interventions can have lasting impacts on rumen microbial diversity and function, potentially enhancing adaptation to forage-based diets and improving energy metabolism [57,58,59].

Collectively, the temporal and diet-driven shifts in community diversity suggest that rumen microbial communities respond to nutritional interventions over time, providing insight into how early-life nutritional strategies may influence microbiome development for sustainable, high-performing livestock systems. Increases in alpha diversity are thought to confer greater metabolic flexibility and resilience to dietary changes [59], and enhanced microbial diversity has been associated with improved fiber digestion, volatile fatty acid production, and overall animal performance in ruminants [58]. The patterns observed in the present study are also consistent with findings from supplementation studies in other ruminant and non-ruminant species, where dietary additives such as MRF have been associated with changes in bacterial diversity [17,60,61].

Beta diversity analysis further highlighted time as a major factor in shaping community structure, but also indicated the contribution of diet. The influence of maternal MRF supplementation was most evident in lambs that did not receive MRF directly (Con_Con and MRF_Con), where PERMANOVA analysis indicated that, among lambs never supplemented with MRF directly, maternal supplementation status explained a greater proportion of beta diversity variation (Figure 5). This observation is consistent with the idea that maternal diet may influence the rumen environment, influencing the trajectory of microbial community assembly in offspring [8,62,63,64]. This preconditioning could occur through multiple mechanisms, including direct microbial transfer, alterations in milk composition, environmental exposure, or other maternal effects [7,65,66]. Our findings are in agreement with recent studies showing that dietary interventions, particularly those introduced early in life, can have persistent effects on the rumen microbiome, increasing microbial diversity and potentially enhancing animal productivity and health [2,67]. In this context, both direct and maternal supplementation may contribute to shaping rumen microbial communities during development, with potentially complementary effects on alpha and beta diversity.

The relationship between rumen microbiome diversity (as measured by alpha diversity) and TVFA production is of interest because TVFAs are crucial end-products of microbial fermentation, directly impacting lamb nutrition and productivity [4]. In our study, higher alpha diversity was associated with higher relative abundances of valerate and lower relative abundances of butyrate (Figure 6A). These correlations were strongest at week 8, a period of rapid rumen development and dietary transition (Figure 6B). At week 20, fewer correlations reached statistical significance, although similar directional trends were still observed, particularly for valerate and butyrate. Collectively, these results suggest that greater microbial diversity in the developing rumen is associated with different TVFA profiles, including relative shifts towards propionate and valerate and lower proportions of butyrate and isobutyrate. Such fermentation patterns have been linked in previous studies to differences in methane production and host energy metabolism, as propionate serves as a key precursor for gluconeogenesis [68,69,70,71,72]. In addition, higher valerate levels have been linked to greater protein fermentation potential [73], which could be related to microbial diversity and improved cross-feeding [74].

Rumen microbial composition differed in MRF-supplemented groups, particularly enriching taxa associated with succinate metabolism, a key pathway for propionate production (Figure 7). Specifically, we observed an enrichment of *Succiniclasticum ruminis* at week 20, along with transient enrichment of *Succinovibrio dextrinosolvens*, *Succinomonas amylolytica*, and *Schwartzia succinivorans* at week 8. These observations are consistent with a model in which MRF may promote the proliferation of microbial communities capable of converting succinate to propionate. This observation is consistent with previous studies identifying *Succiniclasticum* as a keystone propionate producer with improved feed efficiency in ruminants [75,76,77]. The coexistence of diverse succinate-metabolizing bacteria (e.g., *Fibrobacter succinogenes*) may contribute to functional redundancy within the rumen, potentially supporting metabolic stability despite temporal variation in individual taxa [78,79]. While these interpretations are supported by the literature, it is important to note that functional activity was not directly measured in this study; future work integrating metatranscriptomics, metabolomics, or flux analyses will be necessary to confirm these functional roles.

The results of this study support a potential mechanistic pathway linking MRF supplementation to host performance via modulation of the rumen microbiome and fermentation profiles. Specifically, MRF supplementation was associated with enrichment of microbial taxa involved in carbohydrate fermentation and succinate metabolism, key intermediates in the production of propionate. This is consistent with the observed increases in propionate and valerate concentrations in supplemented groups. Propionate is a major glucogenic precursor in ruminants and plays a central role in host energy metabolism, providing a plausible link between microbiome composition and animal performance. Although differences in growth performance were not statistically significant, lambs receiving both maternal and direct MRF supplementation exhibited numerically higher average daily gain and final body weight. Together, these findings suggest that early-life MRF supplementation may influence rumen microbial ecology and fermentation pathways in a manner that supports improved metabolic efficiency, although further work is required to confirm causality.

Further analysis of co-occurring microbial clusters within the rumen identified a succinate-focused group, characterized by positive correlations between *Ruminococcaceae* and *Fibrobacter* (succinate producers) and *Selenomonas* (a succinate consumer). This succinate-metabolizing group negatively correlated with a lactate-/amino-acid-focused group, including *Mitsuokella*, *Lactomicrobium*, *Megasphaera*, *Dialister*, *Acidaminococcus*, and *Eubacterium*, which are involved in lactate and amino acid metabolism. A negative correlation between these two groups may reflect potential competition for pyruvate, a key metabolic intermediate in both succinate and lactate production pathways [80]. Such partitioning may contribute to rumen pH stability by balancing the accumulation of lactate (which can lower pH) with succinate-driven propionate production (which is pH-neutral) [81]. For example, *Megasphaera* consumes lactate to produce propionate, thereby mitigating the risk of acidosis, while *Succiniclasticum* directly channels succinate into propionate [78,82].

Although MRF supplementation was associated with changes in the rumen microbiome, these shifts were not accompanied by statistically significant differences in average daily gain (ADG) or final weight. However, the MRF_MRF group consistently exhibited the highest median weight, suggesting potential, albeit non-statistical, advantage. This pattern is consistent with recent findings in dairy goat kids fed MRF-based diets, which reported statistically significant greater body weight, ADG, and dry matter intake [17]. Differences in weight may be partly explained by metabolic partitioning, where propionate (a glucogenic VFA) supports lean tissue growth via hepatic gluconeogenesis [72], while acetate (a lipogenic VFA) is more closely associated with fat synthesis and storage [83,84]. Thus, the observed differences in propionate production may contribute to variation in feed efficiency without necessarily affecting total weight gain. Another explanation for the absence of measurable growth responses may be a temporal lag, as microbial shifts, such as the enrichment of *Succiniclasticum* at week 8, may require longer-term persistence before translating into host-level performance outcomes. Importantly, the observed alterations in rumen microbial composition and fermentation profiles suggest that MRF supplementation may be contributing to biological effects that are not immediately reflected in growth metrics. Variation in the abundance of functionally important microbial taxa and associated fermentation characteristics, such as enhanced fiber-degrading capacity and volatile fatty acid production, may support rumen functional maturation, metabolic efficiency, and resilience to dietary transitions. These microbial and fermentative adaptations could influence animal health, feed efficiency, and robustness later in life, particularly under environmental or nutritional challenges, even in the absence of short-term growth responses. Additionally, sample variability may be a contributing factor; field trials are inherently prone to individual animal variation, which can obscure subtle treatment effects, necessitating larger sample sizes to achieve statistical power [85].

The observed enrichment of succinate-metabolizing taxa in MRF-fed lambs suggests that dietary interventions may influence rumen fermentation towards greater propionate production, a pathway associated with improved animal productivity via gluconeogenesis and lower methane emissions. Nevertheless, several of the taxa identified in this study are known to contribute to key carbohydrate fermentation pathways in the rumen, suggesting potential functional implications that warrant further investigation. However, confirmation of these functional roles would require targeted functional approaches, such as metagenomics with robust annotation, metatranscriptomics, or metabolomics. However, the absence of significant growth responses underscores the complexity of host–microbe interactions, where functional redundancy and metabolic trade-offs can buffer against simple phenotypic changes.

A major limitation of this study on MRF supplementation is the lack of functional analyses (e.g., metatranscriptomics or metabolomics); without these, we cannot confirm whether microbial compositional differences are directly related to metabolic outcomes. To strengthen these findings, future work must prioritize functional validation of microbial pathways (e.g., quantifying propionate/succinate flux), temporal and dose–response analyses to map causality and larger field trials with robust statistical power. Though the observed microbial dynamics align with established metabolic principles (e.g., succinate pathways in rumen efficiency), the absence of functional data leaves mechanistic connections speculative and underscores the need for multi-omics integration in livestock microbiome studies. In the future, a multidisciplinary approach combining microbiome, metabolomic, meat quality, and performance data will provide a holistic understanding of how prebiotics influence lamb growth, health, and product quality, ultimately guiding more effective and sustainable supplementation strategies. Microbiome and fermentation analyses were conducted using a single rumen liquid-phase sample per animal to avoid repeated measures, and information on animal lineage and pen assignment was not available for inclusion as covariates. While this approach reduced model complexity, future studies incorporating mixed-effects modeling and additional animal- and environment-level metadata would strengthen causal inference.

Second, maternal MRF supplementation was a central component of the experimental design; however, samples from ewes (e.g., rumen, milk microbiome, or milk composition) were not collected. Consequently, the mechanisms underlying maternal effects, such as microbial transfer, altered milk composition and oligosaccharides, or other maternal influences, could not be directly evaluated and remain speculative.

Future research should address these limitations through a more integrated and longitudinal approach. Specifically, combining microbiome profiling with functional analyses, such as metatranscriptomics, metabolomics, and targeted quantification of fermentation intermediates (e.g., succinate and propionate flux), would enable stronger linkage between microbial composition and metabolic function. Additionally, dose–response studies and temporal sampling across key developmental stages would help clarify causal relationships between MRF supplementation, microbiome development, and host performance. Finally, larger-scale production trials incorporating animal performance, health metrics, and product quality traits (e.g., carcass and meat characteristics) will be essential to evaluate the practical relevance of MRF supplementation under commercial conditions. Together, these approaches would provide a more comprehensive understanding of how early-life nutritional interventions shape rumen function and long-term animal performance.

## 5. Conclusions

This study demonstrates that maternal and early-life supplementation with a mannan-rich fraction (MRF) can influence the development of the lamb rumen microbiome, particularly during key stages of microbial establishment. The observed shifts in microbial diversity and composition, including enrichment of taxa associated with succinate metabolism, suggest that dietary MRF supplementation may alter fermentation pathways within the developing rumen. These microbiome changes were associated with shifts in fermentation profiles, indicating a potential link between microbial community structure and host energy metabolism. While growth performance differences were not statistically significant, the consistent numerical trends observed in lambs receiving both maternal and direct supplementation suggest potential biological relevance.

Overall, these findings support the use of maternal and early-life prebiotic supplementation as a strategy to beneficially shape the developing rumen microbiome. Although mechanistic links and long-term performance outcomes require further investigation, this work contributes to a growing body of evidence that targeted nutritional strategies may be used to beneficially modulate rumen microbial development and fermentation patterns in young ruminants. Moreover, it will advance our understanding of the complex interactions between prebiotics, the rumen microbiome, and host metabolism, potentially leading to novel approaches for enhancing ruminant production that reduce reliance on antimicrobials and mitigate environmental impact.

## Figures and Tables

**Table 1:** Raw material and chemical composition of the ewe and lamb concentrates.

	Ewe (C)	Ewe (MRF)	Lamb (C)	Lamb (MRF)
Raw Materials (g/kg)				
Barley	225	224	224	225
Wheat Feed	209	209	209	209
Beet Pulp Nuts	100	100	100	100
H/P Sunflower Ext 38	100	100	75	75
Hipro Soya Ext (GM)	99	98	80	80
Soya Hulls (GM)			75	75
Wheat	93	93	61	61
Maise Distillers	70	71	66	65
Cane Molasses	60	60	60	60
Limestone Flour	17	17	15	15
Sodium Chloride	10	10		
Megalac	9	9	13	13
Calcified Magnesite	5	5		
Sodium Chloride			13	13
HJL Sheep Bag 6899	3	3		
HJLO Lamb Txl 0003481			6	6
DCp 18%			6	3
MRF		1		1
Chemical Composition (g/kg dry matter)				
Dry Matter (g/kg)	873	873	874	874
Crude Protein	206	206	194	194
Effective Rumen-degradable Protein (0.05 ^1^)	133	133	120	120
Digestible Undegraded Protein (0.05 ^1^)	55	55	51	51
Oil A	46	46	46	46
Ash	92	92	92	92
Neutral Detergent Fiber	233	233	267	267
Starch and Sugar	344	344	319	319
Metabolizable Energy (MJ/kg dry matter)	14.2	14.2	14.2	14.2

^1^ Fractional outflow rate (kp = 0.05 h^−1^).

**Table 2:** Gaussian-Generalized Linear Model (GLM) of Shannon alpha diversity modeled against volatile fatty acids (VFAs) for all paired liquid samples, at week 8 and week 20.

VFA	Time	Estimate	Std. Error	Statistic	*p*-Value
AC	Week 8	0.002	0.014	0.164	0.871
	Week 20	0.003	0.008	0.424	0.674
Pr	Week 8	0.025	0.014	1.727	0.093
	Week 20	0.006	0.010	0.612	0.545
But	Week 8	−0.070	0.016	−4.279	0.000
	Week 20	−0.027	0.013	−2.065	0.047
Val	Week 8	0.224	0.044	5.089	0.000
	Week 20	0.088	0.044	1.996	0.055
IsBut	Week 8	−0.162	0.151	−1.076	0.289
	Week 20	−0.102	0.175	−0.580	0.566
IsVal	Week 8	−0.058	0.075	−0.776	0.443
	Week 20	−0.054	0.078	−0.693	0.493

## Data Availability

Sequencing data can be found in the NCBI Sequence Read Archive (SRA) database (Bioproject Accession Number: PRJNA1328727). All other data that support the findings of this study are available upon request from the corresponding author.

## Abbreviations

The following abbreviations are used in this manuscript:

ADG	Average daily gain
ANCOM-BC2	Analysis of Compositions of Microbiomes with Bias Correction 2
ANOSIM	Analysis of similarities
BCFA	Branched chain fatty acid
CLR	Centered log ratio
DNA	Deoxyribonucleic acid
GC	Gas chromatography
GLM	Generalized linear model
MRF	Mannan-rich fraction
PCoA	Principal co-ordinate analysis
PERMANOVA	Permutational multivariate analysis of variance
SCFA	Short-chain fatty acid
VFA	Volatile fatty acid
